# Nutritional characteristics of patients with dry mouth and/or Sjögren disease in a Hungarian cohort

**DOI:** 10.3389/fdmed.2026.1879266

**Published:** 2026-08-21

**Authors:** György Tóth, Dorottya Kiss, Csilla Erdei, Krisztina Márton

**Affiliations:** 1 Department of Preclinical Dentistry, Faculty of Dentistry, Semmelweis University, Budapest, Hungary; 2 Department of Oro-Maxillofacial Surgery and Stomatology, Faculty of Dentistry, Semmelweis University, Budapest, Hungary

**Keywords:** dry mouth, hyposalivation, nutrition, Sjögren disease, xerostomia

## Abstract

**Background:**

People with any form of dry mouth may have problems with eating, swallowing, speaking, taste perception, wearing dentures, and maintaining good oral health. Subsequently, it can have a major impact on the quality of life through its effects on the qualitative and quantitative parameters of nutrition. Nevertheless, little is known about the effects of different foods and nutrients on salivation and orofacial sicca symptoms. Despite this, the literature on the relationship between xerostomia (x), hyposalivation (h), Sjögren disease (SD), and nutrition is relatively sparse.

**Objective:**

The authors aimed to evaluate the dietary habits of middle-aged patients with newly diagnosed dry mouth and/or SD compared with healthy controls.

**Methods:**

A total of 128 participants (10 men and 118 women; mean age: 53 ± 13 years) enrolled in the study. A 35-question questionnaire containing a “computer-assisted personal interview”-type exploratory survey was used to record body mass index (BMI), possible food allergies, harmful habits, used sources of health-related information, and nutritional habits such as frequency and types of daily meals, for example, fruit, vegetable, meat, vitamin, and trace element consumption. Subjective sicca symptoms (xerostomia, xerophthalmia) were also assessed by guided questions, while the unstimulated whole saliva (UWS) flow rate was determined by the spitting method to confirm hyposalivation. SD was diagnosed based on the 2016 ACR-EULAR diagnostic criteria. Participants were divided into four groups: healthy controls (c), xerostomia (x), hyposalivation (h), and SD. Patients' data were compared with those of healthy controls.

**Results:**

The mean BMI of the participants did not show a significant difference from the normal BMI, although it was slightly elevated in all four patient groups (c: 24.7; x: 25.4; h: 26.2; SD: 25.2). The smoking percentage was high in the SD group: 49% vs. 18% in the c group (*p* = 0.01), while it was 0 in both the x and h groups. The percentage of food allergy and/or intolerance (lactose, milk protein or gluten, floating, and bloating) was increased among all patients compared with healthy controls: 50% of patients with xerostomia and 44% and 68% with those with hyposalivation compared with only 16% of healthy controls (*p* < 0.05, *p* < 0.05, *p* < 0.00001, respectively). Similarly, liquid consumption was between 1.9 and 2.2 L/day in all groups, and the interviewed participants preferred water ( approximately 78%) and diary milk products (more than 80%). Significantly less patients with xerostomia consumed alcohol (2.6 vs. 18%, *p* < 0.05) and vegetable milk regularly (2.6 vs. 18%, *p* < 0.05) compared with healthy controls; alcohol consumption was also low in the h (4.7%) and SD (6.4%) group of patients. There was no significant difference in the intake of different vitamins and trace elements between any patient group and the c group, except that of vitamin D3, which was consumed in a significantly higher frequency by the x group of patients (*p* < 0.05). Extra sugar, processed food, snack, extra salt consumption, and grain and wheat types of preference were at similar levels in all the examined groups. It was observed that all four groups favored apple among all fruits, while patients with dry mouth did not prefer sour fruits like berries (hyposalivation *p* < 0.05) and citrus fruits (x and h; *p* < 0.05 and *p* < 0.05) or irritating agents like onions (h; *p* < 0.05), as these foods might deteriorate both the dryness feeling and the burning mucosal symptoms. Potato was less preferred by the other groups compared with the healthy control group and might cause swallowing problems in patients of the x group (*p* < 0.05). Nevertheless, rice and tomato-paprika consumption was increased in the h group (*p* < 0.05, *p* < 0.05). With regard to meat types, poultry (50%–88%), pork (40%–58%), and seafood (33%–50%) were the most popular, but possibly because of its less fat content, the x and SD group of patients consumed less poultry compared with the c group of patients (50 and 52% vs. 80%; *p* < 0.001, *p* < 0.05, respectively. It should also be noted that people today prefer the internet as a source for obtaining any diet or health awareness information (64%–92%) and less frequently consult their physicians or specialists (0%–6%). No significant differences were found between the patient and control groups in terms of oil and fat consumption; vegetable oils (82%–100%) and butter (22%–25%) were the most popular between the groups.

**Conclusions:**

As a primary outcome of this study, the nutrition of patients with dry mouth and/or SD generally does not differ from the diet of healthy persons, and it is determined by local traditions and availability. The number of daily meals and the pattern of fluid consumption do not vary significantly because of sicca symptoms or SD. A higher incidence of food allergy was reported in patients with dry mouth and/or SD. Affected patients tend to prefer easily swallowable, non-irritant, softer (rice), or high-water content foods such as tomato and paprika, or cold cuts. Patients with dry mouth and/or SD need conscious dietary advice from their family practitioners, dentists, or specialists to alleviate their symptoms, while trustworthy websites and social media may serve as important sources of health information.

## Introduction

Dry mouth is one of the most frequent symptoms in the population, with a rate of prevalence of 25%–34% (1, 2). The subjective sensation, xerostomia, is primarily a result of inadequate salivary gland function and consequent reduction in the unstimulated whole saliva (UWS) flow rate (3), but it is known that in a number of patients, xerostomia is not accompanied by reduced salivation (4). Hyposalivation, which is objectively verifiable dry mouth, is defined as a condition where the UWS flow rate is ≤ 0.1 mL/min with an estimated prevalence rate of 11%–18%. At the same time, a reduced level may be characterized by a flow rate of ≤ 0.2 mL/min, with a prevalence rate of 17%–22% (5).

Sjögren disease (SD) is a systemic autoimmune disease characterized by dryness of the mouth and/or dryness of the eyes, typical circulating autoantibodies in the serum [Anti-Ro-SSA, Anti-La-SSB, antinuclear autoantibody (ANA) or Rheuma factor (Rf)], and focal lymphocytic inflammation of the salivary and lacrimal glands. Systemic symptoms may include arthritis, primary biliary cirrhosis, and thyroiditis. Currently, the diagnosis is set up according to 2016 American College of Rheumatology/European League Against Rheumatism classification criteria for primary Sjögren disease (ACR-EULAR, 2016) (6), which are based on the weighted sum of five items: anti-SSA/Ro (or others previously mentioned) antibody positivity and focal lymphocytic sialadenitis with a focus score of ≥1 foci/4 mm^2^, each scoring 3, an abnormal ocular staining score of ≥5 (or van Bijsterveld score of ≥4), a Schirmer's test result of ≤5 mm/5 min, and an unstimulated salivary flow rate of ≤0.1 mL/min, all scoring 1. Persons with signs and/or symptoms indicative of SD who have a total score of ≥4 for the items meet the criteria for primary SD. Sicca syndrome, on the other hand, is characterized by subjective and/or objective oral (xerostomia and/or hyposalivation) and/or eye sicca symptoms (xerophthalmia and/or keratoconjunctivitis sicca) without immunoserological or other systemic signs or focal infiltration of the salivary glands.

People with any form of dry mouth may have additional intra- and extraoral sicca symptoms such as burning sensation of the oral mucosa, glossodynia, and problems with eating (taste sensation – dysgeusia, swallowing – dysphagia) (7), which can modify dietary patterns and thereby impact nutritional status. A significant number of patients often report pharyngeal or gastroesophageal reflux disease (GERD) (8). The vulnerable mucosa is prone to injury, ulceration, frequent oral infections, increased Candida and Lactobacillus counts, and increased susceptibility to caries and periodontitis (9). These patients may avoid crunchy foods such as raw vegetables, dry or hard foods such as meats and breads, and sticky foods such as peanut butter. It has also been observed that reduced saliva leads to enzyme deficiency, and saliva is important for digestion of carbohydrates. Xerostomia causes sugars to adhere to teeth (forming dental plaque), increasing bacterial growth and the risk of tooth decay. Poor oral hygiene is an additional challenge, prompting patients to choose softer, easier-to-chew foods (5, 10). Results from a study of the Lithuanian population showed that frequent consumption of carbohydrates, proteins, and oils was less associated with the presence of xerostomia, while people with dry mouth consumed more cold-pressed oils, lean or fatty fish, and probiotic supplements but less breads and processed meats. Esophageal and gastric mucosal lesions associated with sicca symptoms can also cause other general symptoms (11), affecting eating and nutrient absorption. Nutrition-related health awareness is important, and lifestyle can highly influence oral health and diet-related quality of life in persons with dry mouth. A health-conscious person can contribute greatly to the normal maintenance of their oral flora by eating well (12). Dietary factors, on the other hand, may contribute to the etiology and progression of autoimmune diseases such as SD with various sicca symptoms (e.g., dry mouth and dry eyes), and changes in the diet may modify the severity of these disorders (13–15).

Carbohydrates, fats, and proteins are the essential components of a normal diet (16), and carbohydrate intake plays a significant role in oral health and in caries formation in dry mouth conditions. Poor oral hygiene and the presence of missing and/or diseased teeth may lead individuals to prefer softer, carbohydrate-rich foods (17). Nevertheless, the previously cited Lithuanian study indicates that individuals with xerostomia reported a significantly lower consumption of bread, which resulted in an overall carbohydrate intake below the recommended level, leading to a long-term carbohydrate deficit. Severe dry mouth symptoms have also been associated with reduced intake of whole grain products, including certain carbohydrate-containing foods such as cereals, rice, and pasta (11). Proteins are vital foods that are necessary for growth and development, tissue repair, and the production of metabolic and digestive enzymes and certain hormones. Protein loss can be caused by malnutrition resulting from a dryness-induced inflammation of the oral mucosa (18).

Lipids influence the process of adhesion to the palate and the composition and ultrastructure of the primary oral bacterial coat and pellicle. Lipids may also exert anti-inflammatory effects on oral soft tissues and reduce the risk of caries because of their ability to coat tooth enamel (19). Dairy products such as milk, cheese, and yoghurt may therefore offer some protection against tooth demineralization. Regular consumption of dairy products may also reduce the risk of dental caries in the elderly. Milk has similar properties to saliva and contains calcium, vitamin D3, phosphates, riboflavin, B-complex vitamins, and vitamin A. In addition, casein, found in milk, strengthens the enamel (20).

Good nutritional practices and optimal nutritional status promote growth and tissue development and play a major role in disease prevention. Malnutrition, which is associated with a concomitant deficiency of antioxidant micronutrients, promotes underfunctioning of the salivary glands, weakened immunity, and an early shift in the microbial ecology of the oral cavity toward a predominance of anaerobic organisms. Immunosuppression, which includes impaired cytokine function and a reduction in the acute phase response to infection, may prolong periodontal disease (11).

Vitamins D3 and B12, folic acid, and/or iron deficiency may cause orofacial sicca symptoms such as dry mouth, migrant glossitis, and burning mouth and further associated orofacial symptoms such as mucosal atrophy or inflammation. Vitamin deficiencies, on the other hand, may expand the xerostomia-related unpleasant conditions. Patients with autoimmune diseases may have a declined level of vitamin D3 and show a correlation with disease activity in systemic lupus erythematosus and rheumatoid arthritis. According to a previous study, in SD-patients the serum D3 vitamin and the iron levels were significantly decreased. In this same study, patients with hyposalivation, showed a reduced iron level too, compared to the healthy controls (21).

Dry mouth can therefore have a major impact on the quality of life of an individual, not only through sicca symptoms but also through its effects on the qualitative and quantitative parameters of nutrition. Despite this, the literature on the relationship between xerostomia, hyposalivation, SD, and nutrition is relatively sparse: there are very few comprehensive, cross-sectional and follow-up studies of individual nutrients, trace elements, and vitamins (22).

## Objectives

Based on these findings, the authors aimed to determine the UWS flow rate and whether there is a difference, and to what extent, in the dietary habits of people with dry mouth and/or SD compared with those of healthy controls. As part of the primary outcomes of this study, the authors aimed to determine whether the main nutritional parameters of people with dry mouth and/or Sjögren disease differed from healthy controls in terms of BMI, number of daily meals, average fluid intake, preferred types, and consumption frequency of different meals. As part of secondary outcomes, the authors aimed to examine data on participants' preferences regarding the different food types and the sources of health-conscious information.

## Materials and methods

This cross-sectional observational exploratory study included 10 men and 118 women with an average age of 53 ± 13 years. Ninety patients were referred to the “Xerostomia Clinic” Working Group of the Faculty of Dentistry, Semmelweis University (Budapest, Hungary), who visited the institution between 1 February 2021 and 31 January 2023. Patients with subjective dry eyes and/or dry mouth complaints were referred for verification of SD from the Central Hungarian Region (Budapest and its vicinity, urban and rural regions) by their general practitioners and specialists either from the immunology or internal medicine or ophthalmology departments, from the Department of Rheumatology and Clinical Immunology (Semmelweis University, Budapest, Hungary), and from the “Irgalmasrendi” Hospital in Buda (Budapest, Hungary). The control group consisted of 38 age- and sex-matched healthy volunteers from the Education Centre of the Faculty of Dentistry of Semmelweis University (Budapest, Hungary), who otherwise came for routine dental control.

This study was approved by the Scientific and Research Ethics Committee of Semmelweis University (TUKEB license number 315/2021). Participants were informed in writing and verbally about the study details before each session, and they provided their written informed consent.

The diagnosis of SD was based on the 2016 ACR-EULAR classification system. In addition to the subjective sicca symptoms, the diagnosis relies on objective symptoms such as focal lymphocytic sialadenitis, confirmed keratoconjunctivitis sicca (by Schirmer’s test and corneal epithelial staining), hyposalivation confirmed by sialometry, and anti-SSA/Ro autoantibody and/or ANA and/or Rf positivity (6).

### Questionnaire on oral sicca symptoms

The primary study criterion for patients with SD was the confirmation of at least one symptom of dry mouth or dry eyes, and the study participants were referred to by either of these subjective signs. To determine this criterion, the questionnaire included questions on the following: (1) whether the eyes stung as if they had sand in them; (2) whether the patients had persistent dry eyes for more than 3 months, with daily complaints; (3) whether they drank fluids frequently when eating dry foods; (4) whether they used artificial tears more than three times a day. For further testing, at least one positive answer to these questions was required. The exclusion criteria were as follows: active hepatitis C infection, radiotherapy of the head and neck region, sarcoidosis, graft versus host disease, IgG4-related disease, amyloidosis, and acquired immunodeficiency. This questionnaire was also used for participants in the control group.

### Measurement of the unstimulated whole saliva flow rate

Verification of possible hyposalivation was performed by measuring the UWS flow rate through the spitting method (sialometry). Saliva was collected into preweighed vessels between 9.00 and 11.00 am for 5 min, with the patient seated in an upright rested position and expectorating all the formed saliva into the vessel. Patients were asked to refrain from smoking, eating, and drinking 2 h prior to the test session and to avoid swallowing and make as few movements as possible during the procedure (23). For normosalivation, the value was ≥0.3 mL/min, whereas for hyposalivation, it was ≤0.1 mL/min; decreased salivation was confirmed when UWS ≤ 0.2 mL/min (5).

### Exploratory questionnaire to assess the relationship between dry mouth and nutrition

In addition to the personal data of the participants, their physical parameters, eating habits, harmful habits (alcohol consumption and smoking), and complaints were recorded using a “computer-assisted personal interview”-type questionnaire containing 35 questions that were prepared and validated following the method of Baâdoudi et al. (24) and specifically adapted to the special purpose of this study (Table 1). The diet assessment was based on quantitative frequency data in terms of the number of daily meals, amount of daily liquid intake, and the consumption rate of vegetables, fruits, meat, organic food, sugar, salt, fast food, snack, or processed food. In addition to the confessed amounts, to the question, “How often do you eat …?”, four answers could be indicated: (1) never, (2) rarely – less than weekly, (3) often – weekly, and (4) regulary – daily. Through this question, the meaning was indicated to the subjects: never means the person does not prefer the food or drink; rarely means the person consumes the food or drink less than weekly; often means the person consumes the food or drink at least weekly; and regularly means that the person consumes the food or drink daily.

**Table 1:** Questionnaire on the dietary habits of patients with dry mouth and/or Sjögren disease.

I. Personal data, composition parameters, and habits of patients
1. A. Sex? male/female Please sign! B. Age? …years. Please write your correct age in numbers!
2. Body mass index (BMI): (Calculated)….
3. Do you smoke daily? Sign, please! Yes/No
4. Do you drink alcohol daily? Sign, please! Yes/No
5. What is your source of health-conscious information (in terms of healthy nutrition, healthy living)? Sign, please! Internet, Newspaper, Journal, Television, Friends, Expert (dietitian, general practitioner, dentist, etc.)
6. Do you have any food allergies and/or intolerance? If yes, to what kind of food? Sign please! Yes/No. Type, food:….

II. Food supplements consumed
7. Do you usually take vitamin C? Sign please! Never, Rarely: less than weekly, Often: weekly, Regularly: daily
8. Do you usually take magnesium? Sign please! Never, Rarely: less than weekly, Often: weekly, Regularly: daily
9. Do you usually consume Omega 3 fatty acids? Sign please! Never, Rarely: less than weekly, Often: weekly, Regularly: daily
10. Do you take any type of vitamin B or vitamin B complex (B6, B12)? Sign please! Never, Rarely: less than weekly, Often: weekly, Regularly: daily
11. Do you usually take vitamin A? Sign please! Never, Rarely: less than weekly, Often: weekly, Regularly: daily
12. Do you usually take vitamin D? Sign please! Never, Rarely: less than weekly, Often: weekly, Regularly: daily
13. Do you usually take vitamin E? Sign please! Never, Rarely: less than weekly, Often: weekly, Regularly: daily
14. Do you usually take a multivitamin complex? Sign please! Never, Rarely: less than weekly, Often: weekly, Regularly: daily
15. Do you usually take zinc tablets? Sign please! Never, Rarely: less than weekly, Often: weekly, Regularly: daily
16. Do you usually take selenium tablets? Sign please! Never, Rarely: less than weekly, Often: weekly, Regularly: daily
17. Do you usually take iron and/or folic acid supplements? Sign please! Never, Rarely: less than weekly, Often: weekly, Regularly: daily

III. Questions regarding eating and drinking habits
18. How many meals do you eat daily? 1, 2, 3, 4, 5, more
19. Do you consume animal milk on a daily/weekly basis? Sign please! Yes/No
20. Do you consume vegetable milk on a daily/weekly basis? Sign please! Yes/No
21. Do you consume probiotic dairy products (kefir, yoghurt, buttermilk, etc.) on a daily/weekly basis? Sign please! Yes/No
22. What kind of fat do you use for cooking and for meals? Sign please! Fat, Vegetable oil, Functional oil, Rapeseed oil, Other:……
23. How often do you eat snacks (chips, cakes, candies, chocolates)? Sign please! Never, Rarely: less than weekly, Often: weekly, Regularly: daily
24. How often do you eat vegetables? Sign please! Never, Rarely: less than weekly, Often: weekly, Regularly: daily
25. What kind of vegetables do you usually eat, raw or cooked, and/or garnish? Please nominate! A. Pumpkins (cucumbers, pumpkins, melons, Pattisson, zucchini, watermelons) B. Legumes (beans, peas) C. Cabbages (Chinese kale, broccoli, cauliflower, kohlrabi, cabbage), D. Root vegetables (horseradish, radish, parsley, carrots), E. Leafy vegetables (spinach, sorrel, lettuce, chicory, arugula, pigeon lettuce, iceberg lettuce), F. Onions (onion, garlic) G. Tomato, paprika (sweet, hot, chilly, Hungarian sharp and hot, bell pepper, etc.) H. Potatoes (potatoes, sweet potatoes) I. Rice (white rice, brown rice, red rice, black rice, wild rice)
26. How often do you eat fruits? Sign please! Never, Rarely: less than weekly, Often: weekly, Regularly: daily
27. What kind of fruits do you usually eat? Please nominate! A. Apples (apple, pear, quince, medlar) B. Dry crop – oily nuts (nut, walnut, cashews, peanut, pistachios) C. Drupaceous (cherry, sour cherry, apricot, peach, plum) D. Berries (strawberry, raspberry, blueberry, blackberry, redberry, hips, currants, grapes) E. Citrus (lemon, lime, orange, tangerine, satsuma, yuzu, bergamot) F. Tropical (banana, avocado, pineapple, kiwi, dates)
28. How often do you eat meat, meat dishes? Sign please! Never, Rarely: less than weekly, Often: weekly, Regularly: daily
29. What kind of meat do you usually eat? Please nominate! A. poultry (chicken, turkey, partridge, rooster, pheasant, etc.) B. pork C. beef D. wild (venison, veal, wild boar, wild rabbit) E. lamb/goat meat F. seafood (fish, lobster, mussels, shrimp, etc.)
30. How often do you eat at fast food restaurants (e.g. Mcdonald's, KFC, and Burger King)? Sign please! Never, Rarely: less than weekly, Often: weekly, Regularly: daily
31. How often do you eat "organic" foods? Sign please! Never, Rarely: less than weekly, Often: weekly, Regularly: daily
32. How often do you add extra traditional sugar (granulated sugar, cane sugar) to your meal (coffee, tea, fruits, etc.)? Sign please! Never, Rarely: less than weekly, Often: weekly, Regularly: daily
33. How often do you add extra salt to your meal? Sign please! Never, Rarely: less than weekly, Often: weekly, Regularly: daily
34. How much liquid do you drink per day (in l)? ….
35. What kind of liquids do you prefer to drink? Please nominate!…

Qualitative frequency data were also assessed in terms of different milk products, liquid, vegetable, fruit, meat, and fat types, dietary supplements such as vitamin A, B, C, D, and E, and trace elements like selenium, zinc, iron, and folic acid. They were tested with the question type: “Do you consume … on a daily/weekly basis?”. A “yes” answer denoted the actual consumption of the given dietary product. Nevertheless, the questionnaire contained both close-ended (multiple-choice and dichotomous questions) and open-ended questions, where the respondents could give their answers in their own words with the assistance of a dietitian (T Gy). Directed questions helped the respondent to form an answer, with the aid of questions about certain habits, types of food, and quantitative data. The answers to the questions were recorded by the person in charge of asking questions (T Gy) using a computer in all cases (25).

### Patient groups

**Figure 1:**
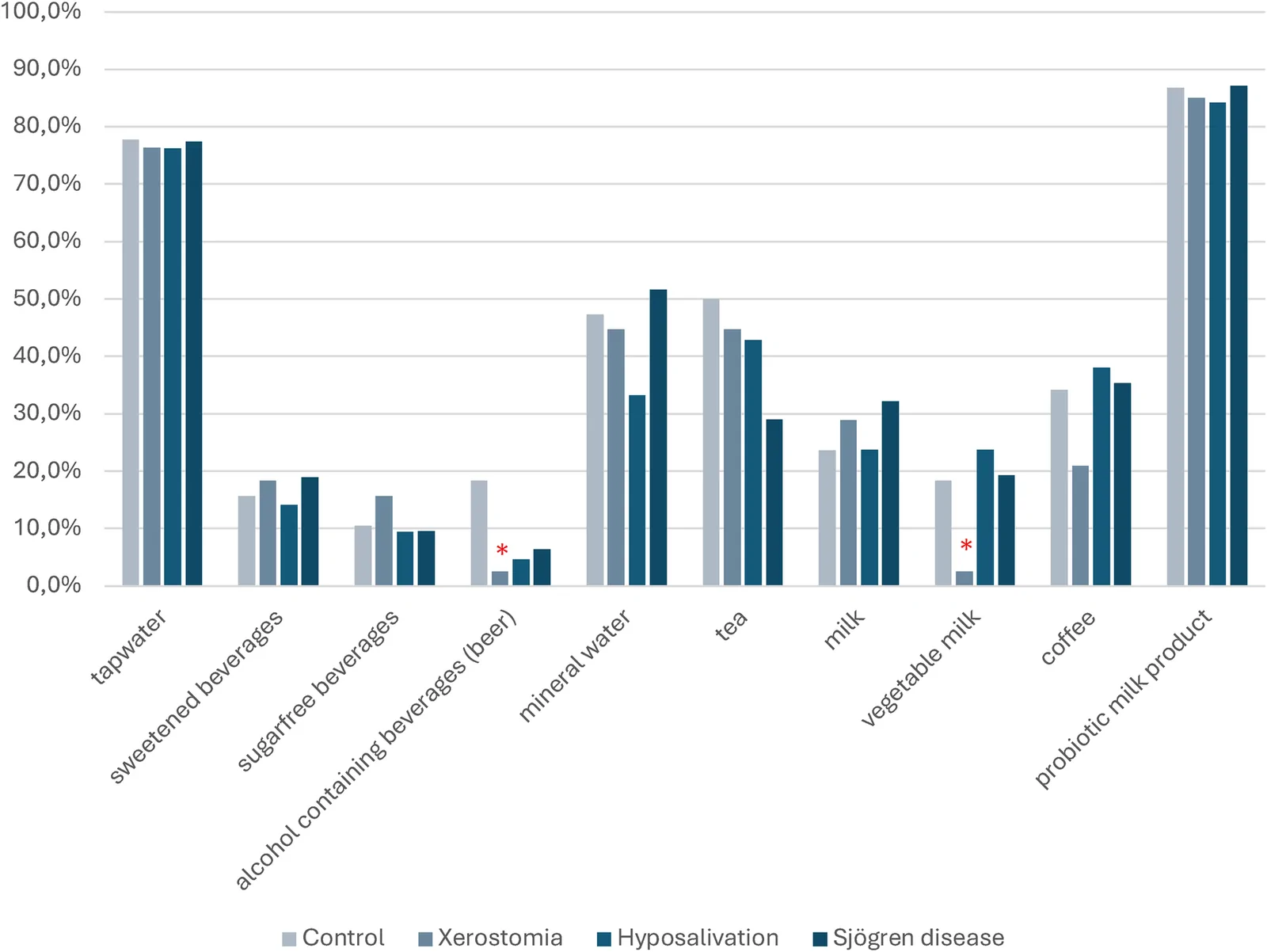
Distribution of different fluid intakes (%) in people with dry mouth and/or Sjögren disease compared with healthy controls. Patients with xerostomia consumed significantly lower amounts of alcoholic beverages and vegetable milk compared with those in the control group (*p < 0.05 by Pearson's chi-square test). No significant differences in the consumption of other types of fluids were found compared with the controls.

Of the 128 subjects who enrolled in the study, four groups were established based on the analysis of the xerostomia questionnaire responses and SD diagnostic results (Figure 1, Table 2):

- Control group (1): healthy individuals without symptoms and complaints (*n* = 38); they did not have any systemic diseases, did not use any medications, and did not show any of the dry mouth symptoms as defined by the “Questionnaire on oral sicca symptoms” used in the diagnosis of SD.
- Xerostomia group (2): patients with xerostomia without hyposalivation and SD (*n* = 38),
- Hyposalivation group (3): confirmed hyposalivation (UWS ≤ 0.1 mL/min) without the presence of SD (*n* = 21),
- SS group (4): confirmed patients with SD (*n* = 31).

**Table 2:** Characteristics of the patient groups with dry mouth and/or Sjögren disease.

Categories	1. Controls (*n* = 38)	2. Xerostomia (*n* = 38)	3. Hyposalivation (*n* = 21)	4. Sjögren disease (*n* = 31)
Age (years) ± SD	48.4 ± 9.57	53.2 ± 14.9	56.7 ± 1.,4	55.6 ± 12.6
Male/female	4/34	3/38	1/20	1/31
Body mass index	24.73 ± 5.65	25.40 ± 7.32	26.29 ± 6.76	25.27 ± 6.32
Liquid consumption/day (l)	2.08 ± 0,54	2.19 ± 0.81	1.84 ± 0.75	1.87 ± 0.72
Ratio of smokers	18.4%	0	0	**47.6% (*p* = 0.01)**
Ratio of food allergy and/or intolerance	15.8%	**44.7% (*p* = 0.03)**	**52.4% (*p* = 0.01)**	**67.7% (*p* = 0.01)**
Number of meals/day	2.81	2.89	2.71	2.87
Unstimulated whole saliva flow rate (mL/min)	0.43 ± 0.21	0.48 ± 0.28	**0.1 ± 0.01 (*p*<0.05)**	**0.24 ± 0.21(*p*<0.05)**
Ratio of reduced salivation (UWS ≤0.2 mL)	21.2%	10.1%	100%	21%
Cardiovascular disease medication	0	8	5	8
Psychiatric disease medication	0	1	1	0
Thyroid gland disease	0	9	1	11
Diabetes	0	1	1	0
Tumor therapy	0	1	0	2
Gastro-oesophageal reflux disease	0	1	0	0

Participants belonged to the middle-aged population with a female predominance.

Ratio of smokers was significantly higher in the SD group, and the UWS flow rate was significantly decreased in the hyposalivation and in the SD group compared to the controls.

Separate comparisons were then made between the control group and the three patient groups. There were no significant differences in terms of gender distribution (based on Pearson's Chi-square test) and age distribution (Student's 2-sample *t*-tests) in the four groups.

### Statistical analysis

Data were presented in the form of mean ± the standard deviation (SD). SPSS 26.0 (IBM, USA) software was used for statistical analysis of all data. Normality was tested by using the Kolmogorov–Smirnov test. Patients' results were individually compared group by group with the outcomes of the healthy controls in order to avoid multiple comparison problems. Factors such as age, sex, body mass index (BMI), liquid consumption, and smoking data were tested by using the ANOVA test. In addition, the Mann–Whitney test was employed for comparing the unstimulated whole saliva flow rate, the number of daily meals, and liquid consumption. Pearson's chi-square test with Fisher's exact correction was used to statistically compare the meal/liquid/ food supplement proportions and frequency between the groups. The significance level for the statistical tests was set as *p* ≤ 0.05.

### Transparency statement

The lead author confirms that this manuscript is an honest, accurate and transparent account of the published study. The report of this work complies with STROBE guidelines. The lead author confirms that no important aspect of the study has been omitted and that any deviations from the study that were planned have been explained.

## Results

### Characteristics of the patient groups

Of the 128 subjects who enrolled in the study, four groups were established based on the analysis of the xerostomia questionnaire responses and SD diagnostic results (Table 2):

- Control group (1): healthy individuals without symptoms and complaints (*n* = 38),
- Xerostomia group (2): patients with xerostomia without hyposalivation and SD (*n* = 38),
- Hyposalivation group (3): confirmed hyposalivation (UWS ≤ 0.1 mL/min) without the presence of SD (*n* = 21),
- SS group (4): confirmed SD patients (*n* = 31).

Comparisons were then made between the control group and the three patient groups separately. There were no significant differences in gender distribution (based on Pearson's Chi-square test) and age distribution (according to the ANOVA test) in the four groups. Participants' associated diseases, UWS flow rates, and other characteristics are visualized in Table 2.

### Body mass index

BMI in the different patient groups was as follows: group 1: 24.73 ± 5.65, group 2: 25.40 ± 7.32, group 3: 26.29 ± 6.76, group 4: 25.27 ± 6.32 (Table 2). In terms of BMI mean values, the patient groups were slightly above the normal range (20–25). There was no significant difference in any of the patient groups compared with controls.

### Duration of daily nutrition intake

In all patient groups, a majority of the participants ate more than three times a day [control (c): 50%, xerostomia (x): 52.6%, hyposalivation (h): 38%, SD: 41.9%] or three times a day (c: 34.2%, x: 42.1%, h: 47.6%, SD: 51.6%; respectively). Two times per day consumption was observed in 13.1%, 5.2%, 14.2%, and 6.4% of the participants, respectively. Only one control subject resorted to one-time daily eating. There were no significant differences between dry mouth/and or SD respondents compared with controls.

### Smoking and salivation

The smoking percentage was much higher in patients with Sjögren disease than in those in the control group (18.4% vs.47.6%, *p* = 0.01 by the Pearson's chi-square test) (Table 2).

With regard to smoking, neither in the control group (non-smoking: 0.45 ± 0.21 vs. smoking: 0.33 ± 0.20, *p* = 0.49 by the Mann–Whitney test) nor in the Sjögren group (non-smoking: 0.26 ± 0.22 vs. smoking: 0.18 ± 0.16, *p* = 0.31 by the Mann–Whitney test) could any significant differences be detected in UWS flow rates.

### Food allergies (lactose, milk protein, gluten) and food intolerance

Forty-four percent of patients in the xerostomia group, 50% in the hyposalivation group, and 68% in the SD group reported food allergy (lactose, milk protein, or gluten) or intolerance (inflating and detached following meals) compared with only 16% of healthy controls, which was significantly less compared with the patient groups (x: *p* < 0.05, h: *p* < 0.01, SD: *p* < 0.01) (Table 2).

### Vitamins and trace elements, omega-3 fatty acid intake

Vitamins D and C were consumed most frequently by the examined patients (daily: 61%–87% and 53%–78%). Of these vitamins, a significantly higher vitamin D3 consumption was confirmed in the case of patients with xerostomia compared with those in the control group (*p < 0.05 based on the Pearson chi-square test) (Table 3). No significant differences were found in the consumption of other vitamins and iron. The daily intake of vitamin D3 in patients with SD was higher than that in the control group patients, and although each patient group consumed more vitamin C than the control group, no significant difference could be confirmed in these consumption patterns (Table 3).

**Table 3:** Proportion (%) of food supplements (vitamins, iron, omega-3 fatty acids, and trace elements) consumed by people with dry mouth and/or Sjögren's disease compared with healthy controls.

Patient groups	Daily	Weekly	Rarely	Never	Daily	Weekly	Rarely	Never	Daily	Weekly	Rarely	Never	Daily	Weekly	Rarely	Never	Daily	Weekly	Rarely	Never	Daily	Weekly	Rarely	Never
a. Vitamins (%)	Vitamin A				Vitamin C				Vitamin B-complex				Vitamin D3				Vitamin E				Multivitamin product			
Control	13	0	11	76	53	0	39	8	24	0	29	47	61	0	26	13*	18	0	24	58	11	0	32	58
Xerostomia	21	0	11	68	67	0	19	14	21	0	47	32	84	0	13	3*	22	0	3	75	17	0	49	34
Hyposalivation	14	0	10	76	76	0	19	5	24	0	38	38	67	0	14	19	10	0	14	76	9	0	55	36
Sjögren's	16	3	10	71	71	0	19	10	19	7	45	29	87	0	3	10	16	0	10	74	9	0	25	66
b. Trace elements (%)	Omega-3 fatty acid				Iron/folic acid				Magnesium				Zinc				Selenium							
Control	32	0	29	40	11	0	32	58	45	0	34	21	18	0	26	55	13	0	19	68				
Xerostomia	34	0	29	37	46	0	24	29	42	0	50	8	40	0	18	42	42	0	16	42				
Hyposalivation	24	0	29	48	14	0	27	59	62	0	14	24	19	0	14	67	24	0	14	62				
Sjögren's	36	0	19	45	44	0	34	22	45	6	26	23	32	0	19	48	26	0	19	55				

Of the vitamins, significantly higher vitamin D3 consumption was confirmed in the case of xerostomia patients compared to the control group (*p < 0.05 based on the Pearson’s chi-square test). No significant differences were found in the consumption of other vitamins and iron. There was no significant difference in the consumption of trace elements between the patient groups examined and the control group.

There was no significant difference in the consumption of trace elements between the patient and the control groups. The hyposalivation group consumed magnesium not significantly more often than the other groups of patients. In terms of zinc consumption, patients with xerostomia and SD consumed at a higher rate of frequency compared with other patient groups, but no significant difference was confirmed (Table 3). Omega-3 fatty acid intake was reported daily in 1/3–1/4 by the examined subjects, and there was no significant difference in the results.

### Amounts and types of daily fluid consumed

The values for fluid consumption were as follows: controls: 2.08 ± 0.54 L, xerostomia: 2.19 ± 0.81 L, hyposalivation: 1.84 ± 0.75 L, SD: 1.87 ± 0.72 L (Table 2). Because the recommended daily fluid intake is 2–2.5 L, it can be concluded that there was no markedly high or low fluid intake among the four groups of patients, with the values close to the recommended intake.

Tap water and mineral water were the main types of drinking water for the subjects. Patients with xerostomia consumed significantly less alcohol containing beverages and vegetable milk products compared with the control group (*p < 0.05 by Pearson's chi-square test) (Figure 1). No significant differences in the consumption of other types of fluids were found between the other groups and controls. All those interviewed preferred tap water: this was mentioned in more than 75% of the participants in the questionnaire, while mineral water and tea was also quite popular (mentioned in 33%–52%). The rate of consumption of sweetened beverages was 15%–19% and that of sugar-free drinks was 10%–15%, while for cow milk and coffee, the rate ranged between 21% and 35% (Figure 1). The rate of overall use of vegetable milk was 2%–24%, while for alcohol intake, it was 2%–18%, according to the results of the questionnaire.

Respondents answered yes to the question on regular alcohol consumption, and the consumption proportions are shown in Figure 1. The highest proportion was reported by the members of the control group (18%), while the lowest proportion was reported by those with hyposalivation (0) (*p* < 0.05).

### Frequency of organic food consumption

**Figure 2:**
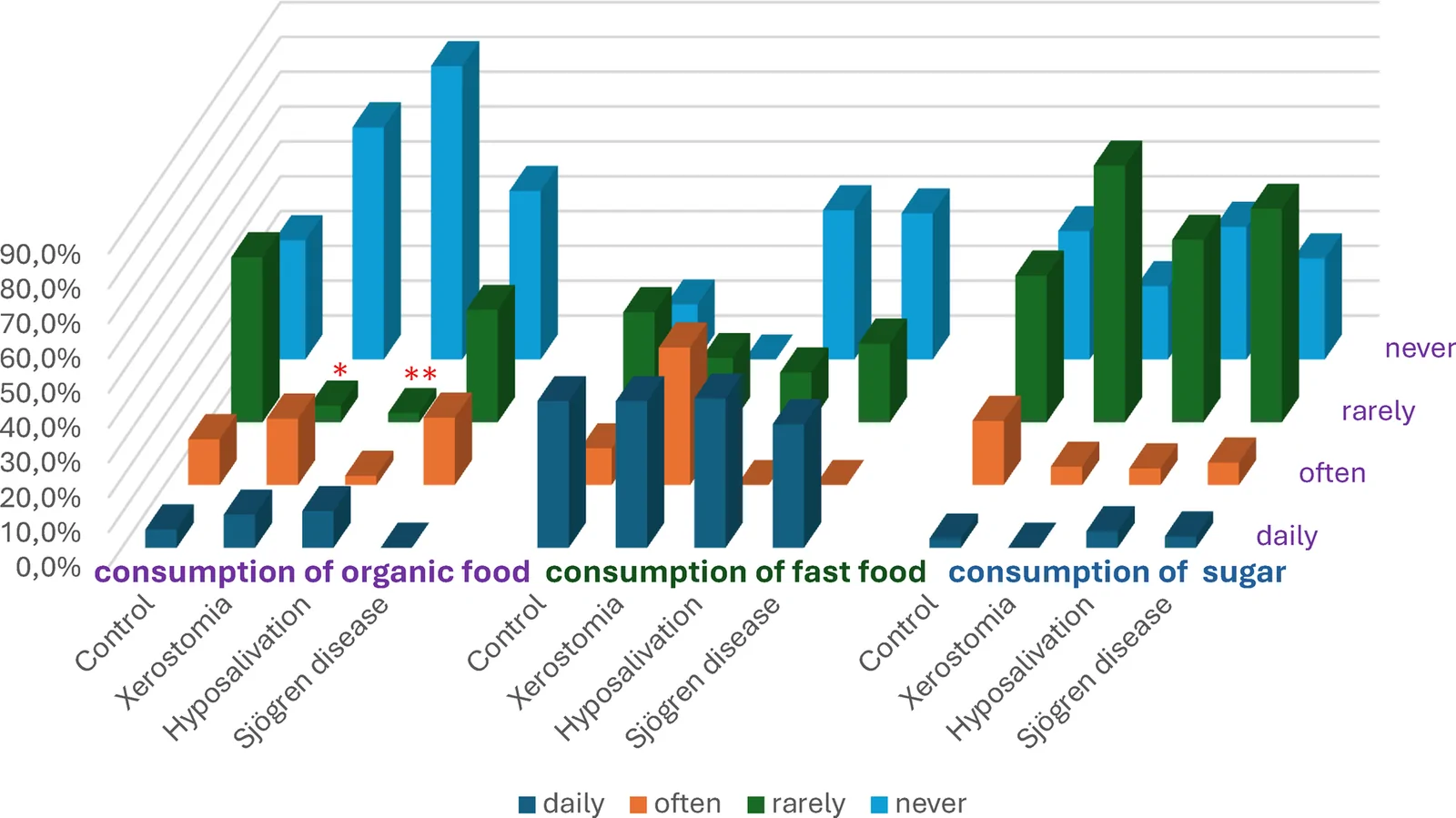
Consumption of organic food, fast food, and sugar in patients with dry mouth and/or Sjögren disease. Patients with xerostomia and hyposalivation reported a significantly lower consumption of organic foods compared with those in the control group (*p < 0.05; **p < 0.00001). There were also no significant differences between the patient and control groups in the consumption of fast food or sugar.

In this studied cohort, the rate of consumption of organic food was low, and the percentage of those who did not consume organic food was higher (34%–84%) than those who did. Patients with xerostomia and hyposalivation consumed much less organic food (*p* < 0.00001 and *p* < 0.05, respectively, as appropriate) compared with the control group of patients, while patients with SD consumed a similar proportion of organic food as the controls (Figure 2).

### Source of gaining health awareness information

**Figure 3:**
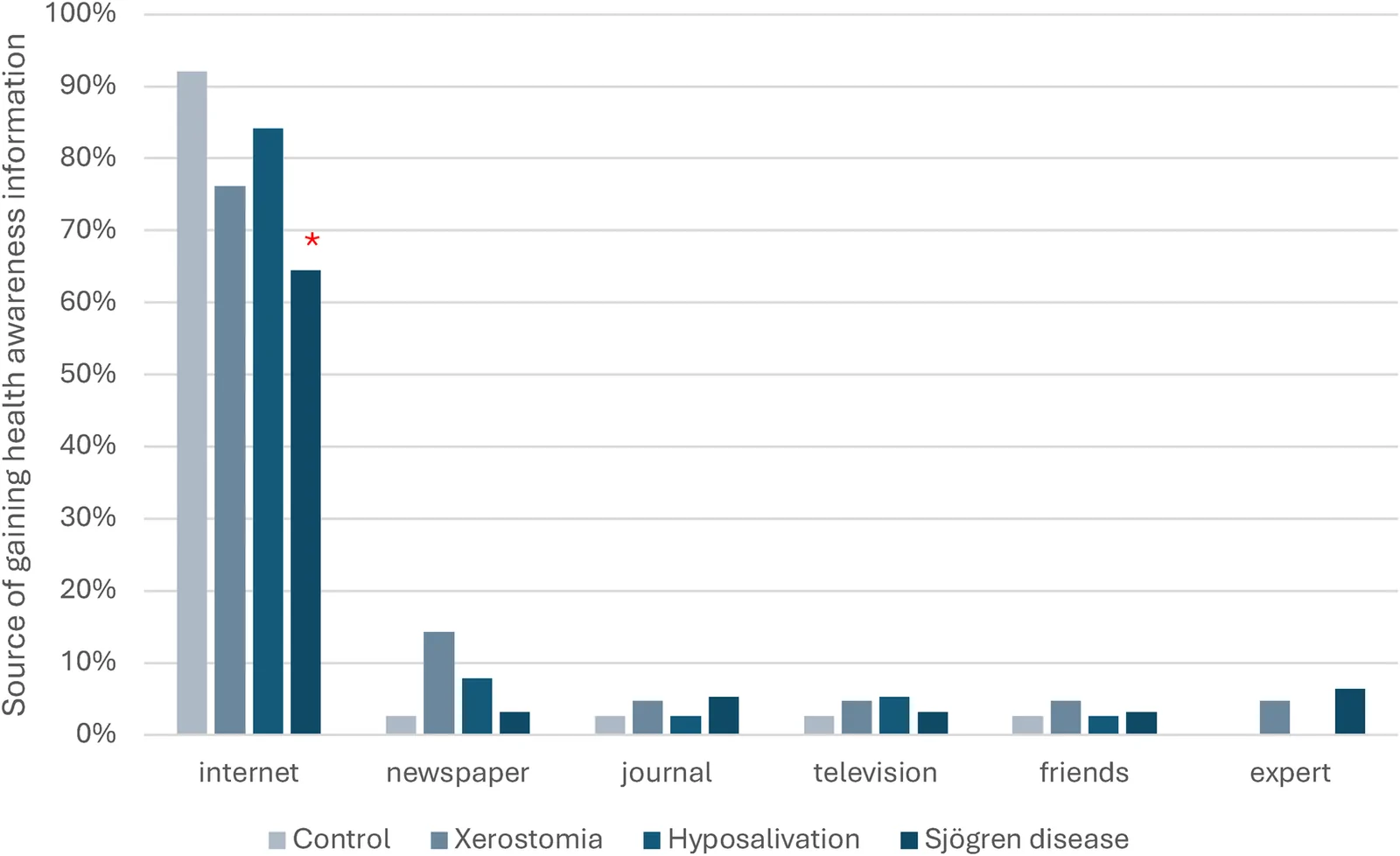
Proportion of sources of health-related information for people with dry mouth and/or Sjögren disease compared with healthy controls. Members of the Sjögren disease patient group reported a significantly lower internet use compared with their control group counterparts (*p < 0.05 based on Pearson's chi-square test). However, 6% or less respondents in the patient groups consulted an expert.

Today, a majority of patients obtain information from the Web (65%–92%), although in this study, the members of the SD group reported a significantly lower internet use compared with those of the control group (* *p* < 0.05 based on Pearson's chi-square test). However, 6% or less than this percentage of respondents in the patient groups consulted an expert. Patients with xerostomia preferred newspapers compared with the other groups of patients, but the difference was not significant (Figure 3).

### Frequency of vegetable and fruit consumption

**Figure 4:**
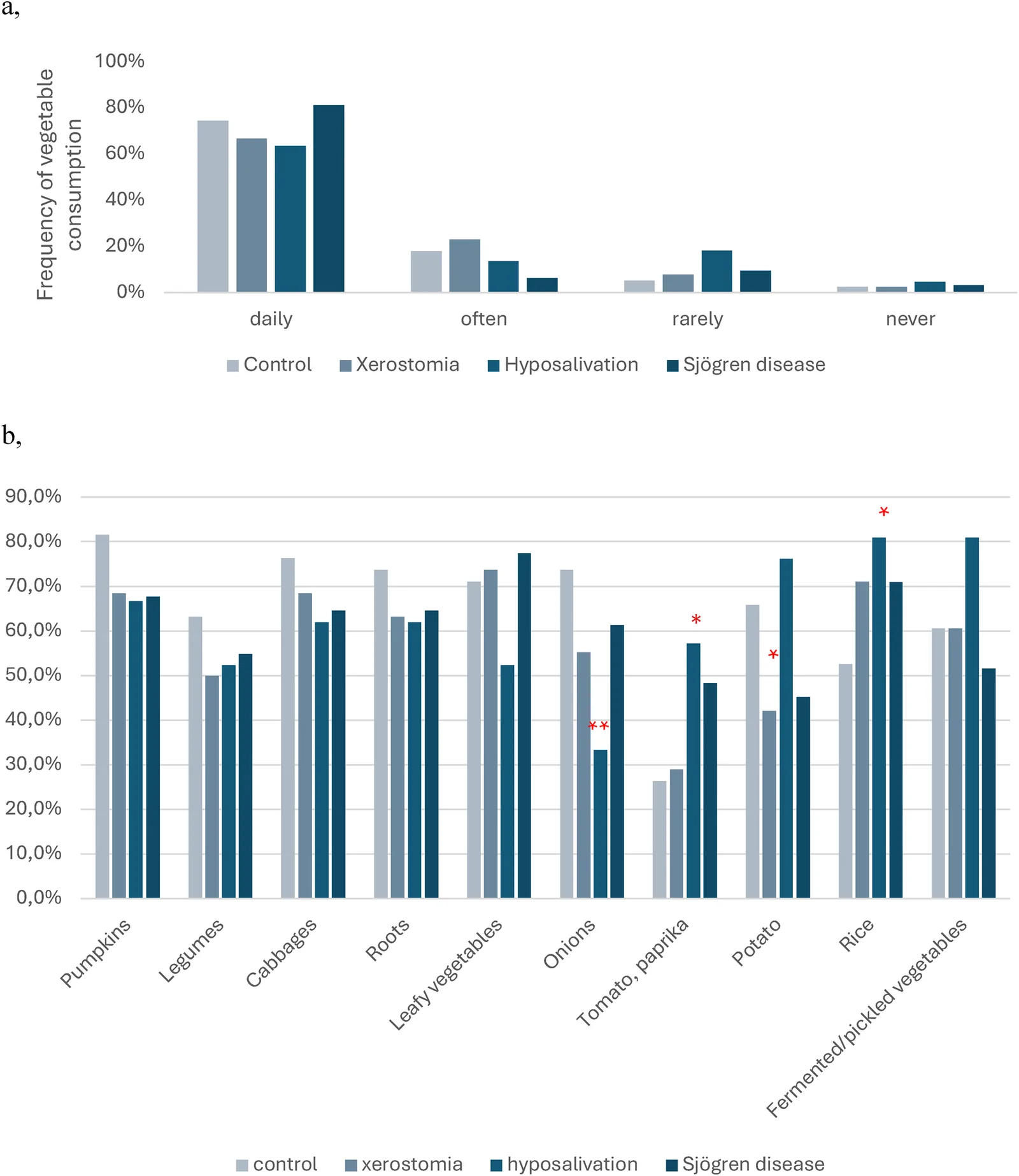
**(a)** Frequency of vegetable consumption, **(b)** types and ratio of preferred vegetables in people with dry mouth and/or Sjögren disease compared with healthy controls. Patients with xerostomia reported a significantly lower consumption of potatoes and patients with hyposalivation reported a significantly higher consumption of rice and tomatoes and paprika and a lower consumption of onions compared with those in the control group (*p < 0.05, based on Pearson's chi-square test) and **p < 0.01, based on the same test)

Subjects of the examined groups preferred a daily consumption of different vegetables (65%–82%), and only a few of them (less than 10%) communicated that they never ate green products. Veggies were similarly popular in all the examined groups, and approximately 30%–80% consumed them. Patients with xerostomia reported a lower consumption of onions (*p* < 0.01) and potatoes (*p* < 0.05) but preferred more tomatoes and paprika (*p* < 0.05, by the Pearson's chi-square test) than the controls. Patients with hyposalivation, on the other hand, preferred an increased amount of rice (*p* < 0.05, by the Pearson's chi-square test) compared with those in the control group. At the same time, there was no significant difference in the consumption of other vegetables between the patient groups (Figures 4a,b).

**Figure 5:**
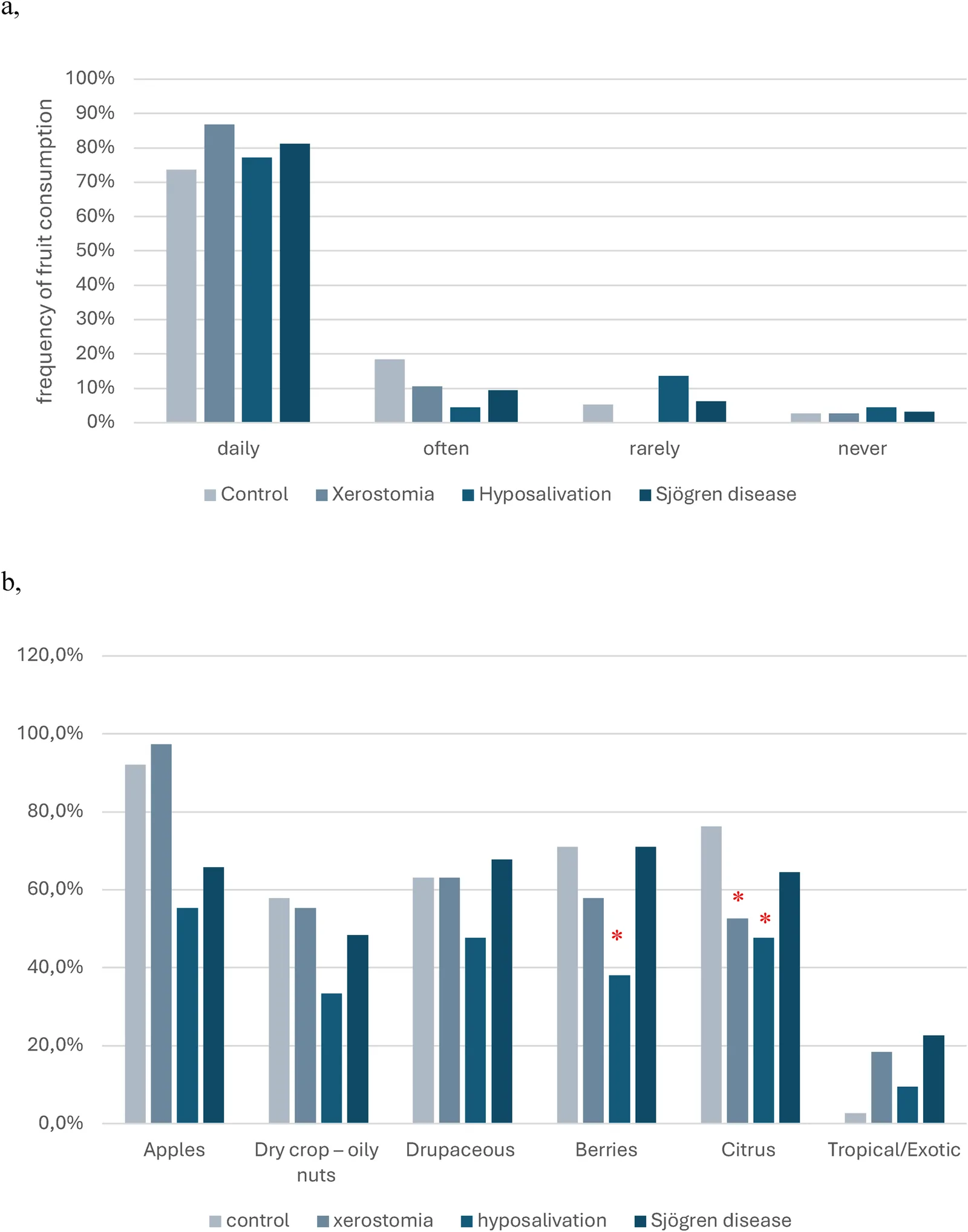
**(a)** Frequency of fruit consumption, **(b)** types and ratio of preferred fruits in people with dry mouth and/or Sjögren disease compared with healthy controls. All four patient groups consumed mostly apple fruits. Patients with hyposalivation consumed less berries and citrus foods; the Sjögren disease patient group reported a significantly lower consumption of citrus foods compared with those in the control group (*p < 0.05 by the Pearson's chi-square test).

Most of the examined persons declared that they ate fruits daily (45%–69%) or often (9%–49%), with people belonging to the category of rarely comprising only 5%–19% and that of never being only 3%–5%. There was no significant difference in the frequency of fruit intake. The preferred fruit types were apples in 80%–100% of the questioned patients, while other types were also popular (38%–78%). People with hyposalivation, on the other hand, consumed less berries and citrus foods, while citrus intake was also reduced in patients with xerostomia (*p* < 0.05 by the Pearson's chi-square test in all the three cases) (Figures 5a,b).

### Consumption of meat products

**Figure 6:**
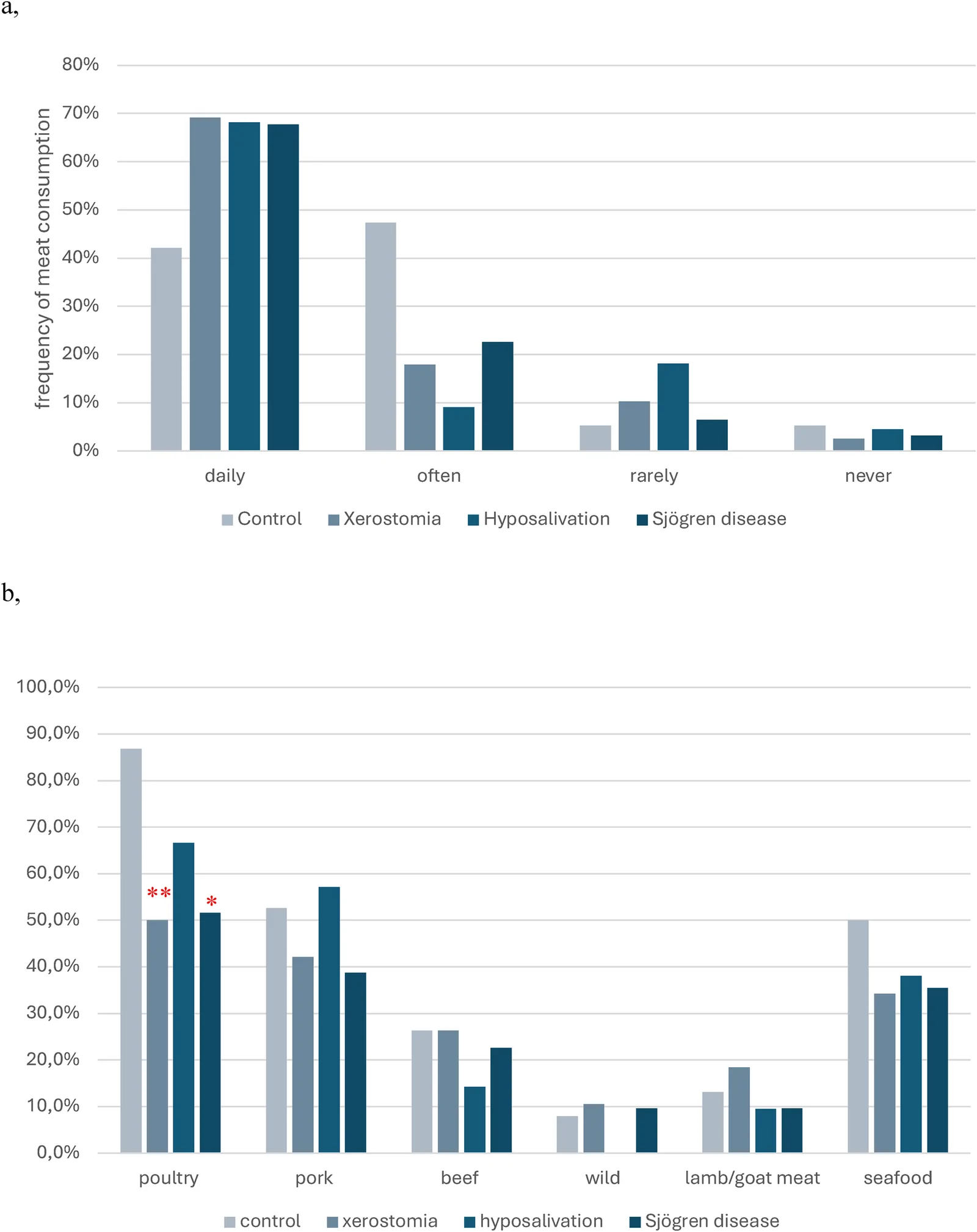
**(a)** Frequency of meat consumption, **(b)** types and ratio of preferred meat types in people with dry mouth and/or Sjögren disease compared with healthy controls. Patients of the xerostomia and Sjögren disease groups consumed significantly less poultry than those in the control group (*p < 0.001, **p < 0.05 by Pearson's chi-square test). There was no significant difference in the consumption of other meat types.

Most of the examined people declared that they ate meat daily (42%–69%) or often (9%–49%), while only a few (3%–19%) of them belonging to the rarely or never category. There was no significant difference in the rates of frequency compared with the controls, although only 42% of the controls mentioned about daily consumption. Subjects preferred poultry (39%–89%) and pork (39%–59%), but seafood was also popular (35%–50%). Other meat types (beef, wild, lamb, and goat) were consumed by 10%–28% of the questioned ones. Members of the xerostomia (*p* < 0.01) and SD groups (*p* < 0.05 by the Pearson's chi-square test) consumed significantly less poultry than the control group. There was no significant difference in the consumption of other meat types by these groups compared with the control group (Figures 6a,b).

### Consumption of fats and oils

**Figure 7:**
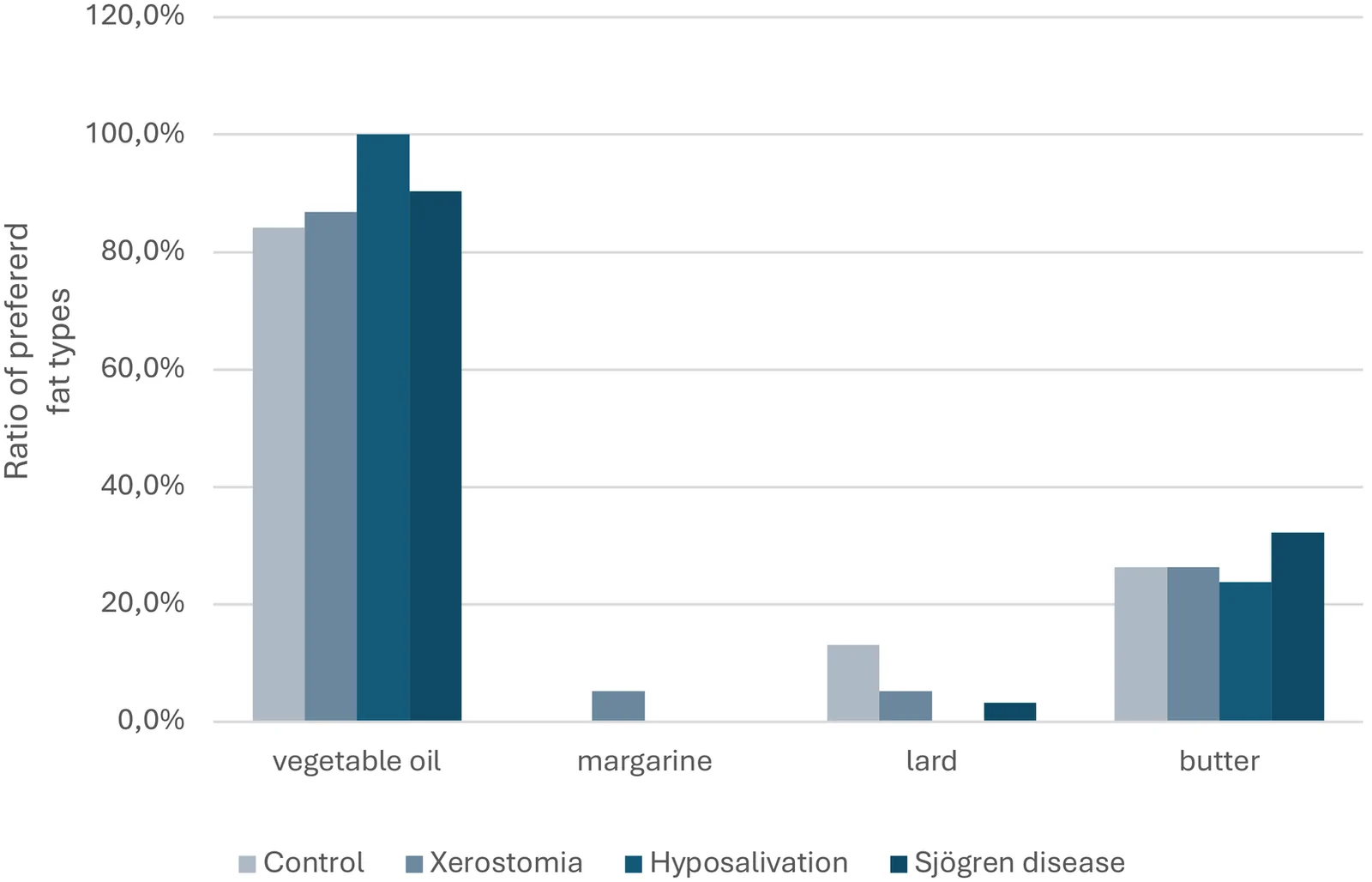
Proportion of used fat and oil types for cooking in people with dry mouth and/or Sjögren disease compared with healthy controls. Most of the questioned subjects preferred vegetable oils and consumed butter in a lesser amount. There was no significant difference in the consumption of the different fat types.

Persons of the examined groups preferred either raw-type vegetable oil or cooking-type oil (81%–100%) and butter (21%–35%). Margarine and lard were mentioned by only 5%–15% of the questioned patients. There was no significant difference in the consumption of the different fat types compared with controls (Figure 7).

### Frequency of consumption of sugar and fast food

Persons of all groups answered rarely (42%–73%) or never (21%–38%) to the question about the frequency of extra sugar intake. Only a few of them answered daily (0%–4.8%) or often (4.18–18.4%). There was no significant difference in the patient groups compared with the healthy control group.

Patients answered rarely (42%–75%) and never (21%–39%) to the question “How frequently do you eat fast food,” and only 3%–19% confessed that they consumed daily or often. Nevertheless, there was no significant difference between the patient and control groups in fast food consumption (Figure 2).

### Wheat consumption

**Figure 8:**
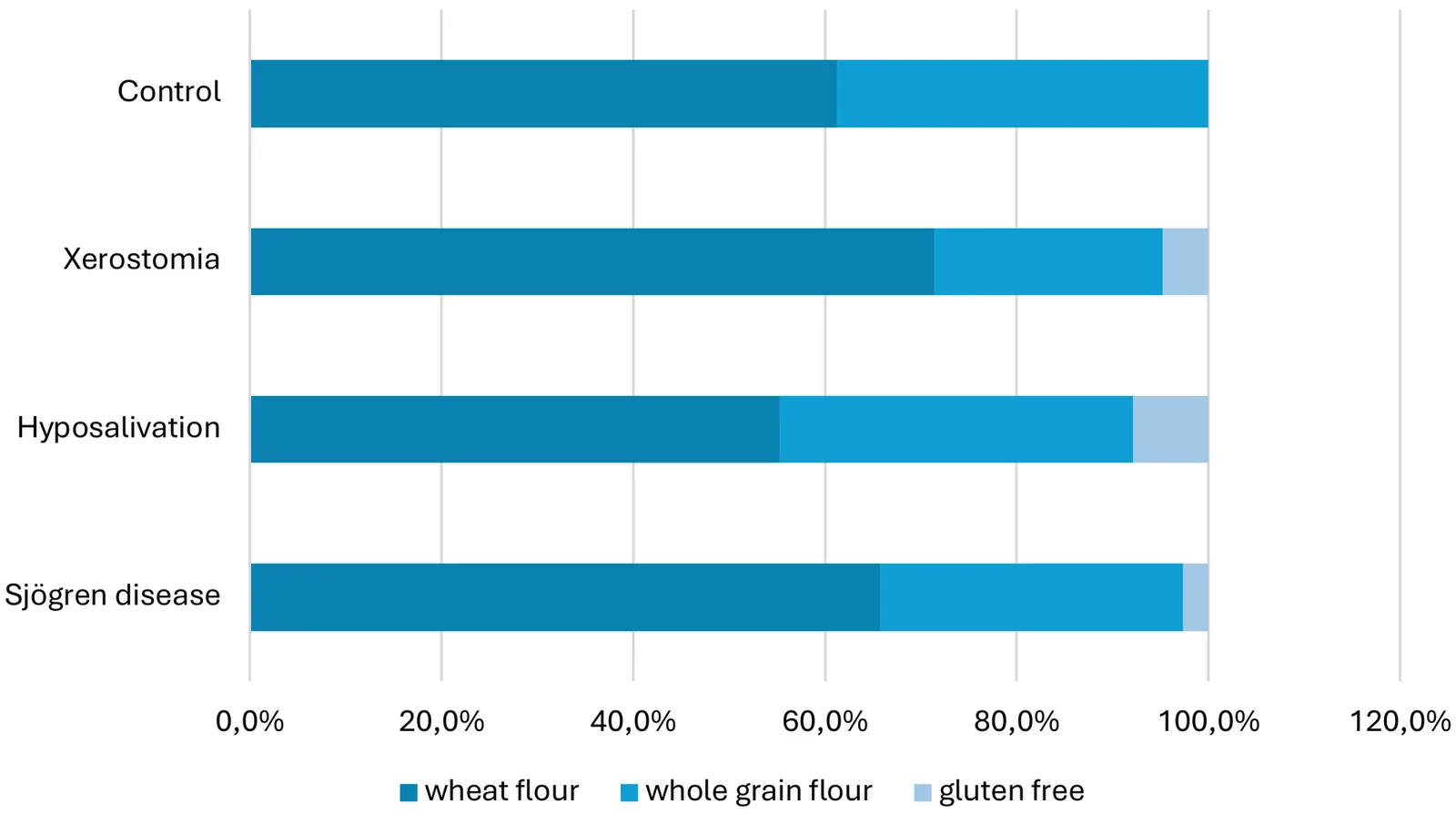
Trends in the frequency of consumption of different wheat and grain types. Wheat flour was the most favored grain type among the patient groups; whole grain was consumed in a lesser amount. A gluten-free diet was mentioned by less than 10% of the questioned patients. There was no significant difference in the patient groups compared with healthy controls in any of the eaten grains or wheats.

According to the obtained data, wheat flour was the favorite grain type among the patient groups (56%–77%), while whole grain was consumed by 25%–39% of the questioned subjects. A gluten-free diet, on the other hand, was mentioned by only 0%–9% of the questioned. Significant differences could not be established in any patient group compared with the control group (Figure 8).

## Discussion

This study was limited to Hungarian patient groups. However, it should also be noted that the pattern of food consumption of these groups reveals and confirms the eating habits of the general population of Central-Eastern Europe. The questionnaire in this study was structured for the specific purpose of determining the nutritional habits and dietetic knowledge of the Hungarian population and was based on these parameters. The results reveal the characteristics of this population; nevertheless, the data can be applied to the food attributes of other European populations because there are some basic nutrients that determine the human diet of Europe and additionally in a limited way, of the whole world.

Sicca symptoms only minimally limited the quality and quantity of nutrients consumed in these examined patient groups. People with orofacial sicca symptoms commonly prefer foods that are easy to chew and swallow, do not cause irritation of the orofacial mucosa, and do not stick to the teeth, gums, or palate. At the same time, it should be noted that financial availability and easy access to different products are also important aspects. Stimulating saliva secretion and keeping the oral mucosa moist to reduce sicca symptoms is an important factor in the “diet.” However, it is important that the chosen food or nutritional supplement also contributes to the maintenance of the health of the oral mucosa and furthermore, the esophageal and gastric mucosa, and finally to systemic health. In addition, the healthy composition of the oral microbiome can be a key factor in this case for the health of the oral cavity, and through it for that of the intestine, and ultimately for that of the individual, as the system of oral antimicrobial defense mechanisms through a dysbiosis can also be disrupted in a dry mouth (26).

### Body mass index and number of daily meals

Considering the body mass index (Table 2), the results showed that the mean values of the patient groups were within the normal or slightly above the normal range, although it must be noted that in the control group 2, in the xerostomia group 2, in the hyposalivation group 6, and in the SD group, there were two female patients whose body mass indices were above 30, and in the xerostomia group 1, the BMI was above 40. In these examined groups, dry mouth and/or SD did not cause significant weight loss or weight gain. There may be several reasons for this: these relatively light sicca signs and symptoms or the applied current treatments may not significantly affect the BMI.

Nesvold et al, who studied the BMI of 20 patients with SD, found that none of the them were underweight. Of them, 12 were of normal weight, 5 were overweight, and 3 were obese; 7 patients had a normal waist circumference, 4 had a moderate waist circumference, and 9 had a significantly higher waist circumference (27).

The number of daily meals did not differ significantly in any of the examined groups; however, the quality and quantity of meals might differ in the daily dietary sequence. This shows that dry mouth and sicca symptoms may not significantly change during the daily sequence and do not cause difficulty in eating.

### Average daily fluid intake and types of fluid

The average daily fluid consumption (Table 2) was similar in the study groups, although it was slightly less than the average daily recommended amount (approximately 2 L/day). Nesvold et al. found that the average fluid consumption was lower in patients with SD (1.7 L/day) compared with the reference group (2 L/day) (27), while increased fluid consumption was observed in patients with severe xerostomia.

With regard to the consumption of fluid types (Figure 1), it was found that all the examined subjects preferred water types and all of them best liked probiotic milk products such as yogurts. Coffee, tea, and milk were also preferred, while patients with xerostomia consumed significantly fewer alcoholic beverages and vegetable milk than the other study groups, although patients with xerostomia complained significantly about food allergy or intolerance. A possible reason for less availability of vegetable drinks may be their unpleasant taste. Alcohol is known as a drying agent, causing consequent pain and irritation of the oral mucosa. Less (but not significant) alcohol consumption in patients with hyposalivation and SD was observed; a few of these subjects liked moderate beer consumption and they felt a positive refreshing effect after consuming this light alcoholic drink. Yet, consumption of high doses of alcohol causes thirst and dry mouth and a decreased production of total protein, amylase, and total electrolytes by the salivary glands. These complications may enhance the existing unpleasant sicca symptoms in hyposalivation (28). The reason for the debilitating effects is not fully understood, but the latest results are attributed to the endocannabinoid system and the renin–angiotensin system through the diuretic effect of alcohol and the binding of its metabolite, acetaldehyde, to central nervous system receptors. Chronic alcoholism also causes dry mouth by increased destruction of salivary gland cells, on the one hand, and by increased apoptosis induced by TNF-α (29), on the other hand.

### Consumption of organic food, allergies, and food intolerances

Figure 2 shows that the lowest levels of organic food consumption were found in patients with xerostomia and hyposalivation. This is unlikely to be related to a lack of health literacy, but rather to financial means. These sources of food are expensive and yet they are rarely available on Hungarian markets.

The reported data revealed that patients with xerostomia, hyposalivation, and SD had a significantly high rate of food allergy and/or food intolerance compared with those in the control group (Table 2). It is also known that GERD is more common in patients with dry mouth (14), but in the present patient groups, only two persons reported GERD. This indicates that food allergy may be a consequence of any type of dry mouth or SD. In their study, Misako et al. found that 292 patients with primary SD had a higher prevalence of food allergy, drug allergy, and associated allergic rhinitis/allergic conjunctivitis than patients with rheumatoid arthritis (30). This may also be related to the altered condition of the mucosa of the gastrointestinal tract, especially mucosal immunity, and changes in oral cavity and alimentary biomarkers (OMICs). Food intolerance, e.g., bloating and a continuous feeling of fullness, may also be related to changes in either the intraoral or the intestinal microbiome (dysbiosis) in various forms of dry mouth.

### Associated conditions and sources of health-conscious information

Medication and other reasons like systemic conditions may cause dry mouth. The examined patients constituted all those who had been referred to the Xerostomia Clinic in the previously mentioned 2 years. Most of them belonged to the middle-aged population and were mainly females, which reflects gender differences in dry mouth and/or SD. Among xerogenic medications, cardiovascular and psychiatric medications, and in two patients, tumor medications, are worthy of mention, while among chronic conditions, thyroid diseases were most prevalent and diabetes was detected in two patients (Table 2).

The responses revealed (Figure 3) that just like the members of the control group, patients in all three groups used the internet for obtaining health-related information. Despite this fact, people with SD used the internet to a significantly lower extent compared with the control group. According to the obtained data, patients did not receive adequate help from their family physicians or from their dentists in terms of dietary or lifestyle advice. It is important to note that the practice of using the internet and obtaining health information is increasing among the people, and those with autoimmune or other diseases often turn to the internet for more information and support. At the same time, it is important to note that unverified information may lead to harmful consequences.

### Fruits, vegetables, meat consumption, and dry mouth

The results (Figure 4a) show that people with hyposalivation, xerostomia, and SD consume as much vegetables as those in the control group. Goldner and Staffier have previously shown that daily fruit and vegetable consumption resulted in improvements in patients with SD: three female patients reported an improvement in systemic lupus erythematosus and SD' symptoms after following a diet with a predominantly raw, plant-based recovery protocol rich in leafy greens, cruciferous vegetables, and omega-3 fatty acids, while eliminating all processed food (31).

Vegetables of all types available in Hungary are preferred by all patient groups, including fermented or pickled greens, which are popular in the Hungarian kitchen. But the results show that vegetables like onion and garlic are less preferred by patients with hyposalivation because they can cause irritation to the body. The possible stickiness of potato may lead to decreased consumption in patients with xerostomia, because in sicca syndromes like dysphagia, it makes swallowing difficult (Figure 4b). Tomato and paprika are more preferred among patients with hyposalivation and also liked by patients with SD, probably because of their high-water content and smooth consistency. Rice is also preferred by patients with hyposalivation because it is easy to chew and swallow, is rich in nutrients, and is neutral to the mucosa. Cucurbits, cabbages, and leafy vegetables are considered important elements in the diet because of their high water content, neutral taste, and smooth consistency that does not irritate the mucosa and are easy to chew and swallow. Therefore, it is important to note that the high water content of vegetables can contribute to enhancing the quality of life of patients with dry mouth. Pedersen et al. studied elderly smokers and found that low fruit and vegetable consumption by them induced less saliva production (32). In addition to their advantageous price, legumes are soft, easy-to-eat nutritious foods when cooked, while melons, for example, are more difficult to access, but at the same time, for patients, they would leave a pleasant taste in the mouth.

Based on the data (Figure 5a), no significant difference in the overall proportion of fruit intake was confirmed among the examined groups. The data revealed that the daily consumption rate was high in all the patient groups. All these lead to the assumption that the examined patients also strive for a healthy lifestyle with their diet. All patients liked apples, most probably because of the high water content, which can alleviate the symptoms of dry mouth. Also, apples are an easily accessible fruit type due to their low price (Figure 5b). Patients with dry mouth, on the other hand, consumed fewer citrus foods and berries, as their acidic juice might cause irritation of the orofacial mucosa as well as tooth sensitivity (33). Increased consumption of citrus foods may increase the risk of erosion of hard tissues, and, therefore, lead to an elevated susceptibility of the affected enamel to caries. Patient awareness should be raised toward these risks. However, it is also worth considering that the relationship between eating habits and disease can be complicated and can be influenced by several factors. Pedersen et al. examined 621 patients to see whether impaired salivary gland function adversely affects fruit and vegetable consumption in older age groups. They concluded that although fruit and vegetable consumption was average, those who had missing teeth or dentures inevitably consumed less fruit and vegetables (32).

Most of the examined persons in this study eat meat daily or frequently (Figure 6a). As the most popular types in the Hungarian kitchen are poultry and pork, the same values are reflected in this study. Although poultry was popular among all participants, it was less preferred among patients with xerostomia and SD (approximately 50%) compared with controls (88%) (Figure 6b). This can be attributed to the fact that poultry can be low in fat, compared with pork and therefore it may cause difficulties in both chewing (dysphagia) and swallowing (dysgeusia) in patients with dry mouth. Pork, because of its higher fat content, may be easier to consume by those with sicca symptoms. Yet, interestingly, seafood was also popular among the questioned people, even though it does not belong to the Central-eastern European kitchen.

### Fat and oil types used

Overall, the study participants used vegetable oil (mainly sunflower oil) and butter the most. Among these, the percentage of patients with hyposalivation who liked vegetable oils was 100 (Figure 7). This is presumably because these fats do not irritate the oral tissues at all. They may even help lubricate the oral mucosa, aiding chewing and swallowing. Additionally, consuming olive oil and other similarly healthy fats may potentially be advantageous in relieving oral sicca symptoms, and it may be worth doing more research to understand how these fats may affect these conditions. Similar results were obtained by Cermak et al. in a study of patients with SD (34). In a previous study, Martín et al. found that the use of olive oil, xylitol, and betaine was effective in reducing dry mouth (35). The study enrolled 40 patients who were treated with radiation therapy for head and neck carcinoma. As a result, the use of toothpaste, gel, and spray products based on olive oil, betaine, and xylitol significantly improved the symptoms of dry mouth and also the quality of life. The use of healthy unsaturated fats, either for cooking or for natural consumption, is therefore important to be recommended for individuals suffering from dry mouth and/or SD.

### Fast food and sugar consumption. Wheat and grain consumption

Most of the participants eat rarely fast food, -according to their admission (Figure 2). The data of this study suggest that fast food or sugar consumption has no significant influence on sicca symptoms.

The data revealed that there was no difference among the examined patient groups regarding the types of flour and grain consumption. Hungarian people presumably selected wheat flour-made white breads and cakes, while a tendency toward healthy nutrition modes showed that even more people chose whole grain flour in their daily menu, with the percentage being 25%–39% (Figure 8). At the same time, a gluten-free diet could not be detected in a higher percentage of people, either in those with dry mouth or in those with SD. However, a significant percentage of patients with xerostomia, hyposalivation, and SD complained about food allergy or intolerance (Table 2). Moreover, it seems, that the type of the consumed grain do not influence the status of dry mouth or other orofacial sicca symptoms.

Extra daily sugar intake was mostly reported rarely or never by the participants. Increased sugar consumption may be particularly harmful to those who suffer from dry mouth. Excessive sugar intake decreases the mouth's normal balance of bacteria, increasing plaque retention and, in turn, the risk of cavities, gum disease, and oral infections. Sticky foods like caramel, gummies, and dried fruits cling to the teeth and gums, prolonging sugar exposure. Dry mouth predisposes to residues, causing sugar to do more damage than usual. Sugary drinks and snacks consumed in excess can lead to dehydration, increasing the symptoms of dry mouth. As a result, with dry mouth, the risk of dehydration increases when consuming excess sugar and drinking sugary drinks. Saliva, which naturally neutralizes acids produced by sugar consumption, is lacking in those with xerostomia, making it harder to manage the effects of sugar (34, 36–38). However, these habits do not appear to be associated with dry mouth or SD in these examined patient groups as intake was low.

### Vitamins and trace elements

No significant difference between the patient groups and controls was confirmed in the intake of vitamin A, vitamin B, iron and folic acid, vitamin C, vitamin E, and omega-3 fatty acid products (Table 3). However, because saliva and tear production decrease in people with SD and the mucous membranes may also be dehydrated, it can be assumed that vitamin A consumption may play an important role in patients with SD. In a study of 25 patients with SD and 13 controls, Szodoray et al. showed that “vitamin A” levels did not differ between patients and controls, yet patients with extraglandular manifestations experienced a significant decrease in vitamin A levels compared with the control group. Vitamin A showed a positive correlation to both NK cells and Th17 cells, while it showed a negative correlation to the values of Schirmer's test. In addition, it was found that fat-soluble vitamins might play an important role in the immune regulatory processes of patients with SD. Therefore, the authors recommended a daily intake of vitamin A: 3,000 μg (39).

Overall, the use of iron products was higher but not significant in the case of people with SD. Nearly 60% of patients with hyposalivation never consume such products, yet a reduced serum iron level may be linked to this symptom (21). Patients with autoimmune diseases and dry mouth require more attention from their specialists regarding serum iron restitution. Vitamin B12 can also be important in the treatment of mucosal symptoms because its deficiency may cause glossitis and dysgeusia as symptoms of pernicious anemia. A daily intake of vitamin B12 (2,000 μg) is suggested for those with SD by Cuny in his study, in which he describes the treatment of a 75-year-old vegetarian patient with vitamin B12 deficiency. In this patient, vitamin B12 intake was found to be successful and to have a cellular regeneration effect of the mucosa during the 20-day treatment, although it did not result in a reduction of xerostomia and its related sicca symptoms (40).

With regard to vitamin C intake, the average daily suggested amount is 1,000 mg/day. Horrobin and Campbell showed by examining the symptoms of SD and sicca signs that vitamin C consumption plays an important role in the synthesis of prostaglandin and helps the activity of the salivary glands (41). In the present study, daily consumption of vitamin C was reported in approximately 70% of patients in the two groups compared with the control group, in which it was approximately 40%.

With regard to the consumption of vitamin D3 (Table 3), it was found that the study participants mainly chose the “daily” or “often” category, suggesting that the majority of them regularly or frequently take vitamin D3. The suggested daily intake is 3,000 μg. Patients with xerostomia consumed vitamin D3 significantly more frequently than their control counterparts. Garcia-Carrasco et al. investigated the immunomodulatory effect of vitamin D3 and found that its deficiency may play a role in the pathogenesis of SD and in the development of lymphomas (42). The results of a previous study (21) also draw attention to the reduced level of, or on occasions, a lack of, vitamin D3 in patients with SD compared with controls. Therefore, the administration of vitamin D3 preparations is important in autoimmune diseases such as SD. Nearly 87% of patients with SD take the vitamin on a daily basis, while the remaining 13% say that they take it only occasionally or never take it at all. However, the relationship between vitamin supplementation and various laboratory signs and sicca symptoms in the examined patient groups requires further investigation. Cuny conveyed in a case report that although low levels of vitamin B12 and iron in a male patient with SD were resolved when a vitamin B12 and iron combination was administered, xerostomia, glossitis, and glossodynia still caused complaints after 12 months, even though the ANA titer did not change. The patient then received a polyvalent immunoglobulin formula (Lactobin®N, 7 g per day) supplemented with oral vitamin D3 (2,000 IU) and, as a result, both sicca symptoms and xerostomia decreased rapidly. After about 2 years, only mild glossitis and upper lip cracking occurred (40).

The daily recommended dose of vitamin E is 300 mg/day, while that of omega-3 is 1,000 mg. Vitamin E and omega-3 fatty acids may have positive effects on the immune system and inflammatory processes of the disease. This is supported by a study by Szodoray et al., who examined 25 patients with SD and 15 healthy controls, and showed that vitamin E had positive effects on interleukin-10 and NK cells by way of reducing the level of inflammatory cytokines in these patients (39).

Castrejón-Morales et al. noted that higher levels of omega-3 consumption correlated to less ocular symptoms and decreased inflammation in the salivary glands and decreased systemic inflammation scores in their 108 patients with SD. They reported that a low level intake of omega-3 fatty acid was common despite its advantageous effects (43), which was also notable in the examined group in the present study. Omega-3 fatty acids can be found in fish food sources such as tuna, salmon, and mackerel (consumed raw or canned), nuts like walnuts, peanuts, peanut butter, and oils like canola, flaxseed, and walnut oil. Vitamin E can be found in foods such as sunflower seeds, almonds, almond butter, avocados, spinach, butternut squash, broccoli, olive oil, trout, and shrimp, to name a few.

In multivitamin consumption (Table 3), no significant difference was confirmed between the examined groups. At the same time, the data suggest that most of the surveyed consume multivitamins rarely or never. This may be because patients do not feel the need to take multivitamins, or they may get vitamins and trace elements from other sources. The authors did not find any literature data on the use of multivitamins in the studied patient groups. It was stated in a recent study that multivitamin supplementation did not result in an advantage in terms of mortality, and although not significantly, the mortality risk was 4% higher among the subjects studied. The authors recommended region-specific eating habits (Okinawa, Sardinia, Nicoya, Costa Rica, Ikaria, Loma Linda) and eating foods high in fiber and vegetables, as well as five fruits per day, along with the consumption of vegetable proteins (44). According to Hungarian rules, vitamins and minerals must be present in significant quantities in dietary supplements. In Hungary, this so-called significant amount means at least 15% according to Article 5 of Regulation (EC) No. 37/2004 and Annex XIII to Regulation (EU) No. 1169/2011. The regulation is quite flexible in terms of the amount of vitamins and minerals, but the regular, long-term consumption of vitamin/mineral products that exceed the recommended daily intake is unnecessary and even undesirable (45).

There was no noticeable difference in zinc and magnesium consumption between the patient groups and the controls (Table 3). The importance of daily zinc intake (25 mg) is supported by a study by Wessels and Rink on the nutritional significance of zinc and vitamin D, which showed that zinc plays a significant role in the regeneration of immune cells, healing processes, and the maintenance of the body's homeostasis. Their study also provides an answer to how zinc deficiency causes deregulated immune responses, as it is essential for DNA replication, RNA transcription, cell division, cell proliferation, and cell activation, especially due to its regulatory role in intracellular signaling (46). On the other hand, Chen et al. showed that hypomagnesemia was the cause of a severe muscle algo spasm and paralysis in a 38-year-old female patient with SD (47). The daily recommended intake of Mg is 250 mg/day.

With regard to the consumption of selenium (Table 3) – with a recommended daily intake of 225 μg – it was shown that, in this study, patients with xerostomia consumed more selenium, although not significantly, compared with the control group. Selenium has important antioxidant and anti-inflammatory properties and may be useful in the treatment of dry mouth (xerostomia), especially in the case of SD. Selenium deficiency was previously studied by Hirsch et al., who examined 107 female patients with SD and 59 patients with axial spondyloarthritis, and later included 11 patients with SD who had polyneuropathies and 15 patients with axial spondyloarthritis. Insufficient selenium intake in people with SD has been found to be associated with severe polyneuropathy, in addition to being associated with Hashimoto's thyroiditis (48). Therefore, selenium is suggested to patients with SD, although such patients in this study were not aware of this trace material.

### Dry mouth and smoking

With regard to smoking, it must be noted that in the Sjögren group, an extremely high percentage of patients could be shown (47.6%). It is also interesting that the patients confessed about this harmful habit. That the authors failed to ask about the frequency of smoking from the patients in the circulated questionnaire may be considered a limitation of this study. Nevertheless, in this SD patient group, smoking did not significantly decrease UWS flow rates. The authors in one of their previous studies examined the possible influence of smoking on xerostomia and salivation (49). This cohort recruited 901 adult persons from four different age groups. The results revealed that a decreased UWS flow rate could be detected only in young heavy smoking females compared with non-smokers, while xerostomia manifested more frequently in the age group of 18–29 years in smoking females and in the age group of 30–39 years in smoking males. Hyposalivation was not correlated to smoking. Nevertheless, dysphagia was more frequent in old (60+ years of age) smoking males and dysgeusia in old (60+ years of age) smoking females. The patients in this study belonged to a younger age group. The role of smoking-related dysgeusia and dysphagia and their contributing factors to nutrition changes need further examination.

Taking all this into account, patients should be made aware of the importance of eating several meals a day, the continuous intake of fluids of quality and in the right amount, the use of oils containing unsaturated fats, and the regular consumption of as many non-corrosive vegetables and fruits as possible daily, that is, those that do not cause irritation to the body, even in a cooked, easy-to-consume state. It is recommended to use probiotic goods from dairy products, and possibly plant-based milk. The consumption of carbohydrates and sugars is allowed only in minimal amounts because of their obviously cariogenic and systemic effects, although naturally originated sweets such as Manuka honey and thyme honey are known to be complementary, safe, and effective therapies for xerostomia, as these agents have shown a significant reduction in both subjective and objective xerostomia (50).

The effect of multivitamin preparations is controversial; however, the control and supplementation of iron, vitamin B12, and vitamin D3 levels is evident and recommended for protecting the mucosa and to reduce sicca symptoms and also for their systemic beneficial effects (21). Trace elements like selenium and magnesium may also have advantageous impacts.

The diet of patients with dry mouth and/or SD may also have an impact on their symptoms; therefore, they require more attention from this point of view. They should receive appropriate nutritional advice from their specialists (dentists and physicians), with the possible involvement of a dietitian. It should also be noted that trustworthy websites and social media pages may serve as important sources of health information and there are several supporting sites like “Sjögren disease and diet” (51). Patients, though, need help to become more familiarized with these channels in order to avoid using false, harmful, untrusted information.

Excluding certain foods can lead to an unbalanced and unhealthy diet and even contribute to malnutrition (52), although symptoms such as malnutrition were not experienced at all by the examined patient population in this study. However, a study on the elderly population (over 65 years of age) that takes into account several other factors (underlying disease, psychosocial situation, condition of dentures, etc.) may reveal symptoms such as malnutrition. Clinical recommendations can help people with xerostomia in alleviating pain, discomfort, dysphagia, and dysgeusia. Appropriate dietary choices can not only help relieve symptoms but also help stimulate saliva production. The recommendations of Müller et al. can be followed in this regard. Future studies should focus on the quantity of nutrient intake in individuals with xerostomia or hyposalivation, in addition to quality, to improve the quality of life associated with oral health and prevent possible malnutrition due to discomfort resulting from sicca symptoms (50).

### Study limitations

The main limitation of this exploratory dietary questionnaire and, in general, questionnaires on dietary recalls or food frequency, is the high susceptibility to subjective biases and memory lapses, which often leads to inaccurate data regarding actual nutrient intake. Participants may find it difficult to remember the exact amount of ingredients consumed throughout the day.

This cross-sectional self-reported study provided limited information, and therefore, the further aim of the authors is the development of a standardized nutrition measurement instrument (a nutrition diary with measurement of consumption) enhanced with laboratory parameters. It may provide a more accurate picture about the precise amount and frequency of intake, which would provide more data in a follow-up study design. A follow-up, together with laboratory parameters, may assist the dietary habits of patients such as those examined in this study.

A further limitation of this study was that the respondents consciously or unconsciously either provided less information or exaggerated their answers. They would have underreported “stigmatized” foods (sweets, alcohol, fast food) and overreported foods perceived as healthy (vegetables, fruits).

This study included new patients with xerostomia who were referred from Central Hungary within a limited time frame of 2 years, and this resulted in a limited number of patients in the different groups. It must be noted, however, that the incidence rate of SD in Hungary is approximately 16% (53). Dry mouth and SD are conditions that involve predominantly females, and therefore, the obtained data in this study are asymmetric from the point of view of gender.

## Conclusion

The primary outcome of this study revealed that the main nutritional parameters of people with dry mouth and/or SD disease did not significantly differ from the healthy ones in terms of BMI, number of daily meals, average fluid intake, and preferred types of meals. The secondary outcome showed minor differences in food preferences. Patients with dry mouth in the three groups ate less berries and citrus fruits and poultry and potato and consume less alcohol, while consuming more paprika, tomato, and rice than those in the control group. Apple, all types of vegetables, vegetable oils, butter, dietary milk products, and water as the mainly preferred drink were popular in all the questioned people. More than half of the people with dry mouth and/or SD had confirmed food allergies and/or intolerance, which could be associated with the altered state of the oral and gastrointestinal mucosa. These patients need conscious dietary advice from their family practitioners, dentists, or specialists to alleviate their symptoms, while trustworthy websites and social media may serve as important sources of health information.

## Data Availability

The raw data supporting the conclusions of this article will be made available by the authors, without undue reservation.
